# Developing a core outcome set (COS) for Dementia with Lewy bodies (DLB)

**DOI:** 10.12688/hrbopenres.13590.2

**Published:** 2023-10-10

**Authors:** Emilia Grycuk, Emily Eichenholtz, Dag Aarsland, Sara Betzhold, Gillian Daly, Rachel Fitzpatrick, Ann-Kristin Folkerts, Elke Kalbe, Joseph PM Kane, Irina Kinchin, Ian J Saldanha, Valerie Smith, John-Paul Taylor, Rachel Thompson, Iracema Leroi

**Affiliations:** 1 Department of Psychiatry, School of Medicine, Trinity College Dublin, Ireland; 2 Trinity College Institute of Neuroscience, Trinity College Dublin, Ireland; 3 Institute of Psychiatry, Psychology and Neuroscience, King’s College, London, UK; 4 School of Medicine, Trinity College Dublin, Ireland; 5 Medical Psychology | Neuropsychology and Gender Studies & Centre for Neuropsychological Diagnostics and Intervention (CeNDI), Faculty of Medicine and University Hospital Cologne, University of Cologne, Germany; 6 Centre for Public Health, Queens University Belfast, Northern Ireland, UK; 7 Global Brain Health Institute, and Centre for Health Policy and Management, Trinity College, Dublin, Ireland; 8 Brown School of Public Health, Brown University, USA; 9 School of Nursing and Midwifery, Trinity College, Dublin, Ireland; 10 Translational and Clinical Research Institute, Newcastle University, UK; 11 Dementia U.K., Aldgate, UK; 12 Lewy Body Society U.K., Wigan, UK; 13 Global Brain Health Institute, and Department of Psychiatry, Trinity College Dublin, Ireland

**Keywords:** Dementia, Dementia with Lewy Bodies, Core Outcome Set, Delphi, Systematic Review, Ageing, Cognition, Memory

## Abstract

**Background:** Dementia with Lewy bodies (DLB) is an important cause of dementia with a range of clinical manifestations, including motor, neuropsychiatric, and autonomic symptoms. Compared with more common forms of dementia such as Alzheimer’s disease, DLB has been the focus of significantly fewer treatment studies, often with diverse outcome measures, making comparison and clinical implementation difficult. A core outcome set (COS) can address this by ensuring that data are comparable, relevant, useful, and usable for making the best healthcare decisions.

**Methods:** Using a multi-stage approach, development of the DLB-COS will include the following stages: (1) A systematic review, following PRISMA guidelines to create an initial long list of outcomes; (2) A two-round online Delphi including clinicians, scientists, policymakers, and individuals with lived experience of DLB and their representatives; (3) An online consensus meeting to agree on the final core list of outcomes (the final DLB-COS) for use in research and clinical practice; (4) A systematic review to identify appropriate measurement instruments for the DLB-COS outcomes; (5) A final consensus meeting of the professional stakeholders who attended the online consensus meeting to agree on the instruments that should be used to measure the outcomes in the DLB-COS; and (6) Global dissemination.

**Discussion:** This is a multi-stage project to develop a COS to be used in treatment trials for DLB. A DLB-COS will ensure the selection of relevant outcomes and will identify the instruments to be used to measure DLB globally.

**Keywords**: Dementia, Dementia with Lewy Bodies, Core Outcome Set, Delphi, Systematic Review, Ageing, Cognition, Memory

## Introduction

Dementia with Lewy Bodies (DLB) and Parkinson’s disease dementia (PDD) together constitute 10–15% of cases, the second most common dementia worldwide ^1^. DLB is characterized by clinical features including cognitive impairment, parkinsonism, visual hallucinations, REM sleep behaviour disorder (RBD), and fluctuations in cognition ^2^. As the global population ages, the prevalence and incidence of DLB is rising, as are the associated healthcare costs ^3^.

Considerably fewer studies are conducted in DLB than in Alzheimer’s disease (AD) and Parkinson’s disease (PD), despite the close biological relationship between these disorders ^4^; fewer specifically investigate interventions or treatments. Comparison of the treatments examined in existing research is complicated by the wide range of clinical outcomes reported ^5^. Digital, mobile, and wearable technology for outcome data collection add to this complex picture, creating methodological heterogeneity in how outcomes are measured. This complexifies evidence synthesis of DLB trial data, precludes rigorous meta-analysis, and weakens translation of evidence into clinical care. A core outcome set (COS) is an agreed standardized set of outcomes that should be measured and reported in all clinical trials. Standardization of clinical trial outcomes supports consistent measurement of patient symptoms, decreases healthcare costs, and minimizes bias.

This project aims to address the methodological heterogeneity in future DLB clinical trials through development and dissemination of a COS for DLB. In developing this, we will consider the number and type of outcomes measured, and the existence of any standardized data collection methods. We will also ascertain the most appropriate outcome rating tool to represent each outcome in the final COS; however, if more than one candidate tool is available, inclusion will be agreed at the level of the consensus meeting.

## Protocol

### Scope

The DLB-COS and the identified measurement instruments are aimed to be used in future research in randomized and non-randomized pharmacological and non-pharmacological intervention studies and in clinical practice. The target population are individuals diagnosed with DLB.

### Design

This study will follow methodological principles developed by COMET and COSMIN and with modifications where necessary ^6– 9^. The DLB-COS was registered with COMET and is publicly available ( https://www.comet-initiative.org/Studies/Details/1963). The study Working Group (WG) will include researchers, clinicians, health economists, and methodologists who will steer and monitor the progress of the study. Any significant changes to the protocol will be communicated to the ethics committee, the journal, and the founders.

This study will involve six distinct stages:

1. Identifying outcomes from a systematic review, and developing a preliminary list
2. Reaching consensus on a preliminary DLB-COS from the perspective of professional and lay stakeholders via two rounds of Delphi surveys;
3. Building on the preliminary list of outcomes (Objective 2), develop a final DLB-COS for use in future DLB research and clinical practice, via an online consensus meeting with professional and lay stakeholders;
4. Identifying and reaching consensus on the most appropriate instruments to measure outcomes in the final DLB-COS, via an online consensus meeting attended by professional stakeholders;
5. Agreeing on the instruments that should be used to measure the outcomes in the DLB-COS via a final consensus meeting of the professional stakeholders who attended the online consensus meeting;
6. Disseminating and promoting the implementation of the DLB-COS globally.

## Phase 1: Stage 1: Protocol design and evidence synthesis through systematic review

We conducted a systematic review, building on the narrative review on outcome measures in DLB trials, led by Rodriguez-Porcel *et al.* ^10^.

Our systematic review examined and synthesized evidence from qualitative and health economics studies, as well as that reported by clinical trials. Eligibility criteria for the systematic review of outcomes used in the DLB literature can be found in Table 1. The review will be prospectively registered and posted at the International Prospective Register of Systematic Reviews (PROSPERO), and can be found at the following link: https://www.crd.york.ac.uk/prospero/display_record.php?RecordID=346808.

**Table 1.** Eligibility criteria for the systematic review of outcomes used in the dementia with Lewy bodies literature.

	Inclusion criteria	Exclusion criteria
**Publication year**	• Any	
**Language**	• No restriction for abstract screening	
**Types of articles**	• Scientific articles published in peer-reviewed journals with available full texts	• Popular articles • Editorials, commentaries etc. • Study protocols • Abstracts only • Conference abstracts • Trial registries
**Study design**	• Comparative clinical trials (regardless of randomization) • Intervention trials with pre-/post assessments regardless of randomization, case reports	• Observational studies • Reviews, meta analysis (for further trials)
**Population/** ** Setting**	• People with dementia with Lewy bodies (DLB) • 18 years and older; all sexes	• Parkinson’s disease (with and without mild cognitive impairment; PD-MCI) • Atypical parkinsonism (e.g., PPS, MSA) • Other dementias (e.g., AD, FTD) • Mixed study samples (e.g., Involving participants with different dementia subtypes) without separate reporting for Individuals with DLB
**Interventions**	Any pharmacological, surgical Or non-pharmacological approach for treating motor and non-motor symptoms in Individuals with DLB at any disease stage and in any setting (e.g., outpatients as well as inpatients)	
**Comparators**	Any: • None • Placebo/Passive control groups/Wait-list • Active control groups (comparison of different pharmacological/ surgical/non-pharmacological interventions)	
**Outcomes**	Any: • Standardized quantitative assessments, including neurological examinations, assessments and tests, and questionnaires (self-and proxy-rating) across all domains, e.g.; ○ Disease severity, motor- and non-motor symptoms ○ Patient-Reported Outcome Measures (PROMs) including quality of life ○ Biomarkers, imaging outcomes ○ Changes in housing and care situation (e.g., institution) • Carer outcomes included in the patient targeted interventions will be considered (cf. Rigby 2021: https://pubmed.ncbi.nlm.nih.) gov/33554912/) • Qualitative approaches to evaluate intervention success across all domains (e.g., outcomes obtained through patient interviews, focus groups) • Economic evaluation outcomes (cost analysis, cost-effectiveness analysis, cost-utility analysis, cost-benefit analysis)	

### Search strategy

The search identified studies through bibliographic databases, trial registers and the grey literature. Bibliographic Databases and trial registers included the following: Medline Ovid (1946-present); EMBASE (1974-present); PsycInfo (1806-present); CINAHL (1981-present); CENTRAL; Web of Science and specific economic databases including NHS EED and EconLit.

Studies were identified using an elaborated search string including keywords regarding the population and the types of studies that will be covered by the COS.

### Study selection and data extraction

Study selection and data extraction will be performed according to PRISMA reporting guidelines.

### Reporting the outcomes

As recommended by Williamson *et al*. ^6^, the outcome matrix recommended by the ORBIT project ^11^ will be used to display the outcomes reported in eligible studies. This can demonstrate the inconsistency of outcomes measured to date and identify potential outcome reporting bias.

## Phase 2: Stage 2: A 2-round online Delphi survey

### Delphi Technique and Design

An e-Delphi survey will be used to reach consensus for the final list of COS for DLB. The outcomes will likely include commonly reported outcomes addressing the key domains affected by DLB: functioning and quality of life, motor and non-motor parkinsonisms, cognitive ability and fluctuations, health economic outcomes, and psychiatric and sleep-related symptoms ^10^. The number of COS items will not be prespecified, but we will adopt a pragmatic approach to ensure that the COS will be clinically useful in a clinical and research context. The survey will be completed by professional and lay stakeholders. Delphi technique is a widely used approach applied to elicit consensus from domain experts regarding real-world knowledge and defined clinical issues for which no previous consensus existed ^12– 14^. This process gathers information from multiple stakeholders while maintaining anonymity and minimizing the challenges of group dynamics among experts ^6, 9, 15– 17^. The administration of e-Delphi usually involves at least two series of questionnaires (referred to as “rounds”), after which structured feedback is provided to all participants. Then, an online or face-to-face meeting takes place to reach consensus ^6, 11^. Even though this component was not a part of the original Delphi process ^11^, it was adopted from the modified e-Delphi procedure, which allowed for experts’ interaction to reach a final consensus ^18, 19^. This method has proven to be effective ^17, 20, 21^.

The e-Delphi will be administered through the DelphiManager software ( http://www.comet-initiative.org/delphimanager/), with each round lasting three weeks and reminders sent at 14 and 18 days. The data from each round will be analyzed and presented to all participants in the subsequent e-Delphi round. Prior to initiation of the first e-Delphi round, the questionnaire will be piloted among work group members as well as lay stakeholders to assess its validity and clarity.

### Delphi panel participant composition and selection

There is no consensus on the recommended sample size for a Delphi study ^7, 22– 24^ and it is common practice to use the existing literature as a guiding example ^23– 26^. We will recruit different groups of lay and professional stakeholders representing the DLB field. We will include enough participants so at least two representatives from each subgroup can attend the consensus meeting ^7, 23, 24^.

### Eligibility

(1) Professional respondents will include geriatric psychiatrists, neurologists, geriatricians, general practitioners, nurses, psychologists, occupational therapists, health economists, researchers, neural engineers, pharmaceutical industry representatives, and representatives of drug regulatory authorities. Should they know other DLB experts, they can nominate them ^27^.

(2) Lay stakeholders will include people with DLB and their care partners or supporters. They will be recruited through relevant civic society organizations.

### Delphi rounds and consensus criteria

Following previous Delphi studies ^17, 23, 24, 28, 29^, survey respondents will assess the importance and meaningfulness of the outcomes. Moreover, following the design adopted by the studies that included people with lived experience of the disease ^22, 26, 30– 32^. At the end of the first e-Delphi round, the participants will have the opportunity to suggest additional outcome domains to be included in the second e-Delphi round (open text option) 6, 9, 22, 24, 26.

### Rating scale

In line with the Grading of Recommendations Assessment, Development, and Evaluation (GRADE) ^33^ and with the RAND appropriateness method ^34^, each outcome will be scored on a 9-point Likert scale, where 1 designates the lowest and 9 the highest score. Overall, scores from 1 to 3 will be defined as ‘not important’, 4 to 6 will be defined as ‘important but not critical’, and scores from 7 to 9 will be defined as ‘critical for inclusion’.

The criteria dictating inclusion of outcomes for both versions will follow the ’70/15’ consensus approach ^6, 23, 24, 28, 29, 35^. This is defined by at least 70% of stakeholders scoring an outcome between 7–9 and less than 15% scoring it between 1–3. Outcomes which receive at least 70% of scores between 1–3 and less than 15% of scores between 7–9 will not be included in the second e-Delphi round. These thresholds are based on the common agreement that the outcomes constituting the final COS are regarded as critical for inclusion, with a clear minority of stakeholders deeming them 'not important' ^6, 35^. Note, outcomes falling under the same domain will be grouped into single outcomes.

## Stage 3: Consensus meeting finalizing LBD-COS

Some participants who complete both e-Delphi rounds will be invited via e-mail to an online consensus meeting, hosted on Zoom. The meeting will aim to have all respondents from the two stakeholder groups. They will discuss, vote, and agree on the final outcomes. Each outcome will be rated according to four additional criteria ^36^:

• Frequency of the outcome in people with DLB;
• Impact of the outcome on people with DLB;
• Preventability/treatability of the outcome;
• Feasibility to address the outcome in clinical practice and research intervention studies.

Consensus will include at least one lay stakeholder voting for inclusion of the outcome, as established by Wuytack *et al.* ^24^.

## Phase 3: Stage 4: Systematic review identifying measurement instruments to be used in the core outcome set

We will undertake a systematic review for the instruments measuring specific outcomes, following COSMIN guidelines ^37^. We will use the following databases: Medline, PubMed, EMBASE, PsycInfo, CINAHL, and CENTRAL. We will use the key sources of measurement in DLB, such as the DIAMOND Lewy toolkit ^38^ and the Movement Disorders Society Recommendations for measurement tools ( https://www.movementdisorders.org/).

### Quality assessment and recommendations

In developing our COS protocol, we will follow the steps outlined by the COSMIN checklist to ensure quality of the procedure ^39^. Recommendations for outcome measurement instruments will be selected based on the preliminary DLB-COS, according to the quality of evidence and feasibility of the outcomes to be used in research and clinical practice. 

## Stage 5: Final consensus meeting finalizing the choice of instruments measuring the core outcome set

A final consensus meeting attended by professional stakeholders will take place to discuss, vote, and agree on the instruments measuring the selected DLB-COS. We aim to select the most appropriate outcome measurement instrument per outcome. Consensus will be defined as at least 70% of participants voting for inclusion of a measurement instrument ^24^.

## Stage 6: Reporting and dissemination

The development of this DLB-COS will be reported based on the Core Outcome Set–Standards for Reporting (COS-STAR) guidelines ^8^.

Our dissemination plan will leverage the wider professional, civic society, and lived experience networks in this area. Professional dissemination will include participation in international Lewy Body conferences and dissemination through the International Alzheimer Association ISTAART PIA group. Dissemination through civic society organisations will include Lewy Body Society (LBS), Lewy Body Ireland (LBI), and Lewy Body Dementia Association (LBDA). They will help us by sharing information on their websites and social media, using accessible language, and highlighting the contribution of PPI and the third sector partners.

The systematic review is registered and posted at the International Prospective Register of Systematic Reviews, (PROSPERO 2022 **CRD42022346808**).

The development of this DLB-COS will be reported based on the Core Outcome Set–Standards for Reporting (COS-STAR) guidelines ^8^.

## Ethical approval

Since we will be working with sensitive information and including people with lived experience in our work, we will apply for ethical approval from the TCD Faculty of Health Sciences Research Ethics Committee. We will also complete a Data Protection Impact Assessment (DPIA), following General Data Protection Regulation (GDPR) guidelines. Professional and lay participants will receive information about the study via email. If they choose to participate, prior to the first e-Delphi round, they will receive and sign a consent form. The e-Delphi responses will be confidential, and participants will have the right to withdraw at any point prior to data analysis.

## Discussion

Currently, no COS for DLB exists. Since there are very few DLB intervention and clinical research studies, a well-developed and globally disseminated DLB-COS for research and clinical use is required. It is imperative that the measured outcomes have relevance to people with DLB and their care partners. This COS will include the views of a wide range of stakeholders. By developing a standardized COS and ensuring that outcomes are measured with appropriate instruments we aim to increase trial efficiency, improve evidence synthesis, reduce research waste, and improve the development of interventions for people with DLB.

However, we acknowledge that the online nature of the survey is a potential limitation in our methods as this may disadvantage people with limited digital access. Nonetheless, increasingly, online Delphi surveys are acceptable and reflect the experience of the majority of stakeholders. We have had to balance the practical elements of completing the surveys and obtaining transnational representation with the need to include under-served groups.

## Study status

At present, our systematic review has been completed and this has been used to develop a long list of outcomes to be rated in our e-Delphi survey. The e-Delphi survey has been composed and is ready to go live once ethical approval is received.

## Figures and Tables

**Table:** 

*Contributor Role*	*Role Definition*
*Conceptualization* *Iracema Leroi*	*Ideas; formulation or evolution of overarching research goals and aims.*
*Data Curation* *N/A*	*Management activities to annotate (produce metadata), scrub data and* * maintain research data (including software code, where it is necessary for* * interpreting the data itself) for initial use and later reuse.*
*Formal Analysis* *N/A*	*Application of statistical, mathematical, computational, or other formal * *techniques to analyze or synthesize study data.*
*Funding Acquisition* *Iracema Leroi*	*Acquisition of the financial support for the project leading to this publication.*
*Investigation* *Ann-Kristin Folkerts* *Irina Kinchin* *Emily Eichenholtz* *Joseph Kane* *Emilia Grycuk* *Sara Betzhold*	*Conducting a research and investigation process, specifically performing the * *experiments, or data/evidence collection.*
*Methodology* *Elke Kalbe* *Dag Aarsland* *Iracema Leroi* *Valerie Smith* *Ian Saldanha* *Federico* *Emilia Grycuk* *Joseph Kane* *Irina Kinchin* *Ann-Kristin Folkerts* *Sara Betzhold*	*Development or design of methodology; creation of models.*
*Project Administration* *Emilia Grycuk* *Gillian Daly* *Emily Eichenholtz* *Rachel Fitzpatrick*	*Management and coordination responsibility for the research activity planning * *and execution.*
*Resources* *https://www.comet-initiative.org/* *delphimanager/contact.html*	*Provision of study materials, reagents, materials, patients, laboratory samples,* * animals, instrumentation, computing resources, or other analysis tools.*
*Software* *https://www.comet-initiative.org/* *delphimanager/contact.html*	*Programming, software development; designing computer programs;* * implementation of the computer code and supporting algorithms; testing of* * existing code components.*
*Supervision* *Iracema Leroi*	*Oversight and leadership responsibility for the research activity planning and* * execution, including mentorship external to the core team.*
*Validation* *NA*	*Verification, whether as a part of the activity or separate, of the overall * *replication/reproducibility of results/experiments and other research outputs.*
*Visualization* *Gillian Daly* *Emily Eichenholtz* *Iracema Leroi* *Joseph Kane*	*Preparation, creation and/or presentation of the published work, specifically * *visualization/data presentation.*
*Writing – Original Draft Preparation* *Emily Eichenholtz* *Iracema Leroi* *Emilia Grycuk* *Joseph Kane*	*Creation and/or presentation of the published work, specifically writing the* * initial draft (including substantive translation).*
*Writing – Review & Editing* *Gillian Daly* *Emily Eichenholtz* *Rachel Fitzpatrick* *Iracema Leroi* *Emilia Grycuk* *Joseph Kane* *Ann-Kristin Folkerts* *Valerie Smith* *Ian Saldanha* *Irina Kinchin* *John-Paul Taylor* *Rachel Thompson* *Sara Betzhold*	*Preparation, creation and/or presentation of the published work by those from* * the original research group, specifically critical review, commentary or revision* *– including pre- or post-publication stages.*

## Data Availability

Underlying data which is relevant to this protocol include the following studies; *Rodriguez-Porcel, F., Wyman-Chick, K.A., Abdelnour Ruiz, C. et al. Clinical outcome measures in dementia with Lewy bodies trials: critique and recommendations. Transl Neurodegener 11, 24 (2022). https://doi.org/10.1186/s40035-022-00299-w* *Patel B, Irwin DJ, Kaufer D, Boeve BF, Taylor A, Armstrong MJ. Outcome Measures for Dementia With Lewy Body Clinical Trials: A Review. Alzheimer Dis Assoc Disord. 2022;36(1):64-72. doi: https://doi.org/10.1097/WAD.0000000000000473* These studies highlight the need for development of a COS in this area.
