# Supplementary material for: Developing a core outcome set (COS) for Dementia with Lewy bodies (DLB)
Source: HRB Open Res. 2023 Oct 10;5:57. Originally published 2022 Aug 18. [Version 2] doi: 10.12688/hrbopenres.13590.2 (PMC9772580; doi:10.12688/hrbopenres.13590.2)
Supplement: Peer review history

# Reviewer report: Reviewer response for version 2

**DOI:** 10.21956/hrbopenres.15045.r36500

**Published:** 2023-11-23

**Authors:** Catriona McDaid

**Affiliations:** 1 Department of Health Sciences, York Trials Unit, University of York, York, UK

No further comments. Thank-you for your considered responses.

Is the study design appropriate for the research question?

Yes

Is the rationale for, and objectives of, the study clearly described?

Yes

Are sufficient details of the methods provided to allow replication by others?

Partly

Are the datasets clearly presented in a useable and accessible format?

Not applicable

Reviewer Expertise:

Applied health research, systematic reviews, RCTs, outcomes

I confirm that I have read this submission and believe that I have an appropriate level of expertise to confirm that it is of an acceptable scientific standard.

# Reviewer report: Reviewer response for version 1

**DOI:** 10.21956/hrbopenres.14846.r33188

**Published:** 2022-12-21

**Authors:** Catriona McDaid

**Affiliations:** 1 Department of Health Sciences, York Trials Unit, University of York, York, UK

This protocol outlines methods for a systematic review to generate a long-list of outcomes for dementia with Lewy bodies, a delphi consensus study to develop an agreed list of outcomes and a literature search (or possibly systematic review), followed by a consensus meeting, to agree a single measurement instrument to assess each outcome. The overall proposed study design is appropriate for the research question, though there is a lack of detail in the reporting in parts.

**Systematic review**

 The systematic review to identify the long-list of outcomes is appropriately registered with PROSPERO. The authors report that the review has now been fully completed therefore it is not helpful to comment on the methods as these cannot be changed. I have reservations about whether it is appropriate to describe this review in the future tense in the paper given it is fully completed. A much briefer account with a link through to the PROSPERO registration may be more appropriate  - I will leave this for editorial direction. If the review is retained then my comments are as follows:

- overall the review is appropriate and a relevant range of databases are searched.
- more detail needs to be added on what the plans were for synthesis of data, in particular the qualitative data - how was it planned to extract this data and what synthesis method was planned to generate from the qualitative data. This could be moved across from the PROSPERO registration only as it would not be approriate to add information post-hoc.
- Table 1 says grey literature was excluded but the search strategy says studies were identified through grey literature.
- excluding study protocols for ongoing studies seems a missed opportunity in terms of currency of outcome included.
- PRISMA is a reporting guideline not a methods guideline so avoid statements such as saying the review methods followed PRISMA.

**Other aspects of design**

- It is good to see the study registered with COMET. The link needs to be checked as I could not get it to work - though I did find the registration by a search of COMET.
- How will outcomes be prepared for presentation in the delphi - is it just a raw list or will there be any consolidation or grouping?
- It is good to see a plan to pilot the delphi survey with the Working Group before circulating it. However there does not appear to be any patients or carers on the Working Group. It is important to pilot the delphi with patients and carers to ensure that the terminology is understandable. 
- It is good to see a pre-specified definition of consensus for the survey and offering the opportunity to suggest new outcomes at round 1. However, there does not appear to be a plan to account for different priorities amongst patients and carers compared to healthcare professionals during the two survey rounds or to report on this. This could be clarified and justification added for the specific approach used.
- Consensus meeting - what is meant by 'proportional representation from the two stakeholder groups'? How will this be determined and what will be done to ensure the patient and carer perspective is adequately represented? Some thought has been given to this in that, at the consensus meeting, consensus will require at least one lay stakeholder voting for inclusion of an outcome. However, this seems quite late on in the process, arguably it is a low bar, and is there a risk of patients important to patients being voted out in the earlier stages? Again justification and rationale for methods used would strengthen the paper.
- 'feasibility to treat the outcome in clinical practice' - how is this being defined and does it include outcomes that could be addressed in non-clinical settings such as social care? 
- Phase 3 Stage 4 - this is described as a literature search in the Abstract and in the section heading in the methods. Yet, in the text it says 'we will undertake a systematic review'. These are not the same thing and this needs clarified. If it is a systematic review I would expect much more detail on the methods. It is not enough just to say that COSMIN guidelines will be followed as these are not entirely prescriptive.
- The statement 'We will conduct a quality assessment of our COS development process using the COSMIN checklist' is unclear.
- The Design section refers to 'six distinct stages' but 5 are listed

Is the study design appropriate for the research question?

Yes

Is the rationale for, and objectives of, the study clearly described?

Yes

Are sufficient details of the methods provided to allow replication by others?

Partly

Are the datasets clearly presented in a useable and accessible format?

Not applicable

Reviewer Expertise:

Applied health research, systematic reviews, RCTs, outcomes

I confirm that I have read this submission and believe that I have an appropriate level of expertise to confirm that it is of an acceptable scientific standard, however I have significant reservations, as outlined above.

# Reviewer report: Reviewer response for version 1

**DOI:** 10.21956/hrbopenres.14846.r33000

**Published:** 2022-11-29

**Authors:** Panagiotis Alexopoulos

**Affiliations:** 1 Department of Psychiatry, Patras University General Hospital, Faculty of Medicine, School of Health Sciences, University of Patras, Patras, Greece

This a study protocol which will contribute to harmonization of studies focusing on the treatment/management of symptoms of DLB. 

 The following comments may help the authors clarify a number of issues. 

- The authors may consider in their systematic review papers focusing also on MCI due to LB. MCI due to AD increasingly attracts scientific attention with regard to cognitive and non-cognitive symptoms as well as biomarkers.
- Even though grey literature belongs to the exclusion criteria, the authors mention that "the search will identify studies through bibliographic databases, trial registers and the grey literature". 
- Taking into account the paradigm change in dementia definitions towards biological defined clinical entities, it is worth considering not only symptoms, but also biomarkers as outcomes. Biomarkers are not established in DLB. Nonetheless, they can serve as useful indirect markers. ( https://www.ncbi.nlm.nih.gov/pmc/articles/PMC5496518/) 
- It is recommended that sport therapists/physiotherapists and speech therapists are included in the Delphi panel. 
- It is not depicted on what criteria the collection of participants for the consensus meeting be based. Will the collection be exclusively based on the proportional representation criterion?
- The authors aim to select only one outcome measurement instrument per outcome. Is this not a reductionist approach, taking into account the different nature of tools assessing the same symptoms/phenomena (e.g. imaging vs. neurochemical markers, self rating scales vs. scales relying on care partner reports)?

Is the study design appropriate for the research question?

Yes

Is the rationale for, and objectives of, the study clearly described?

Yes

Are sufficient details of the methods provided to allow replication by others?

Partly

Are the datasets clearly presented in a useable and accessible format?

Not applicable

Reviewer Expertise:

Mild cognitive impairment, dementia

I confirm that I have read this submission and believe that I have an appropriate level of expertise to confirm that it is of an acceptable scientific standard, however I have significant reservations, as outlined above.

## References

[1] : Diagnosis and management of dementia with Lewy bodies: Fourth consensus report of the DLB Consortium. *Neurology* .2017;89(1) : 10.1212/WNL.0000000000004058 88-100 10.1212/WNL.0000000000004058 28592453 PMC5496518

# Reviewer report: Reviewer response for version 1

**DOI:** 10.21956/hrbopenres.14846.r32799

**Published:** 2022-09-29

**Authors:** Parichita Choudhury, Andrew Ho

**Affiliations:** 1 Banner Sun Health Research Institute, Sun City, AZ, USA; 2 Banner University Medical Center Phoenix, Phoenix, AZ, USA

A number of clinical trials for Dementia with Lewy bodies (DLB) have been completed and more are coming for both disease-modifying therapies and symptomatic therapies. Currently, there are no outcome measures specific to DLB. Previous trials have borrowed from the Alzheimer’s disease or Parkinson’s disease outcome measures. Hence, developing a core outcome set for Dementia with Lewy Body is a high-yield undertaking and is essential for patients who live with this disease, as well as their practitioners and care partners who support them. In this article, Grycuk *et al. *provide an overview of a protocol aimed at developing core outcome measures, which encompasses the varied phenotypes and presentations of DLB.

 The design of this study, in which a questionnaire is formed from the current literature and then placed through two rounds of consensus is an efficient and effective version of the Delphi method for obtaining a comprehensive set of the most impactful symptoms in DLB. Starting with an open-ended questionnaire, a common form of the Delphi method, can lead to superfluous and irrelevant topics, thus prolonging the process. One of the major flaws of the Delphi method is retaining participants through the multiple rounds, and so by taking on the research by reviewing the literature themselves, the authors will be effectively reducing one round from the Delphi process that may yield a loss of participants, as well as ensuring that the first round of questionnaire is of high impact. Additionally, the two rounds of consensus, with a consensus percentage of 70%, will not only include researchers, but also patients, practitioners, and care partners, so all involved parties will have a sufficient voice when approaching decisions regarding what is most important in the disease trajectory, therefore leading to outcomes that truly matter to all parties.

 There are some limitations to this method. One limitation is the omission of biomarkers. Biomarkers are becoming an essential aspect of diagnosing neurocognitive disorders including DLB, but this is an acceptable exclusion as time is a limitation, and the inclusion of biomarkers may further complicate the process. Another limitation is regarding the selection of the outcomes themselves for the questionnaire. There was no mention of how many outcomes are intended to be surveyed once placed through the ORBIT matrix. If there is no cap, there may need to be some flexibility to increase the number of rounds needed during the Delphi surveys if question volumes are high. Finally, the implementation methodology (an online Delphi) disadvantages stakeholders from under-represented and socio-economically underprivileged backgrounds.

 The development of core outcome sets for DLB is an important undertaking. The five stages presented to obtain these core outcome sets are concise and deliberate. The protocol in its current form is, therefore, publishable, despite some limitations.

Is the study design appropriate for the research question?

Yes

Is the rationale for, and objectives of, the study clearly described?

Yes

Are sufficient details of the methods provided to allow replication by others?

Partly

Are the datasets clearly presented in a useable and accessible format?

Yes

Reviewer Expertise:

Neurodegenerative disease, Dementia with Lewy bodies, Alzheimer's Disease

We confirm that we have read this submission and believe that we have an appropriate level of expertise to confirm that it is of an acceptable scientific standard.
